# Developing a children’s health risk behaviour prevention program targeting grade 4–7 learners in the western cape, South Africa: a study protocol

**DOI:** 10.1186/s12889-021-10968-6

**Published:** 2021-05-30

**Authors:** Kurt John Daniels, Hamilton Pharaoh

**Affiliations:** Department of Physiotherapy, School of Health Sciences, College of Health Sciences, University of KwaZulu-Natal, Westville Campus, University Drive, Durban, 4001 South Africa

**Keywords:** Health risk behavior, Child, Prevention program, Primary schools

## Abstract

**Background:**

Health risk behaviour among South African youth is a significant public health concern. Despite a societal mind shift to educating the public on the prevention of health risk behaviour, behavioural change is not progressing at the rate needed to influence health risk behaviour positively. The project aims to explore behavioural trends and willingness to engage in risky behaviour among senior primary school children. Secondly, to design a health risk behaviour prevention program which adequately equips senior primary school children with the necessary life skills to alter risk behaviour engagement.

**Methods:**

The study will make use of an intervention mapping framework and a sequential, explanatory mixed methods design. Stratified random probability sampling will be used to select three primary schools in the region. Nonprobability purposive sampling will be used to select the stakeholders participating in the focus group sessions. Data collection consists of five phases with the results of each stage informing the structure and application of the next. Phase 1 - baseline data collection (needs analysis) using the child health risk behaviour survey. Phase 2 - focus group interviews. Phase 3 - a systematic review of the literature for result analysis triangulation. Phase 4 – the development of the child risk behaviour prevention program based on the outcomes of phases 1,2 and 3. Phase 5 - implementation of the program. Descriptive statistics will be used to analyse the quantitative data. Chi-square, ANOVA and multiple regression analyses will be used to predict health risk behaviour engagement. Thematic analysis will be used to analyse qualitative data.

**Discussion:**

To our knowledge, this is the first study that would attempt to establish a health risk behaviour prevention program in youth and young people in South Africa. Overwhelming evidence exists that adolescents engage in risky health behaviour which may potentially negatively impact their lives. This study provides an opportunity to address a gap in the current strategy by developing a prevention program for young people which could later be supported by further booster programs through their adolescents. This project would serve as a baseline prevention program that could assist in the reduction of risky health behaviour among various communities.

**Supplementary Information:**

The online version contains supplementary material available at 10.1186/s12889-021-10968-6.

## Background

Health risk behaviour among South African youth remains a significant public health concern [1]. Despite a societal mind shift to educating the general public on the prevention of health risk behaviour, behavioural change is not progressing at the rate needed to change health risk behaviour positively [2].

According to the World Health Organisation (WHO), health is defined as “a state of complete physical, mental and social well-being” and not only “the absence of disease or infirmity” [3]. Health risk behaviour can be defined as engaging inactivity, with a frequency and intensity, which can be harmful to your health or well-being.

The risky behaviours that adolescents and young people are engaging in are becoming a serious concern from a health perspective [4]. Physiotherapy forms part of health education, which is a combination of learning experiences designed to help individuals and communities improve their health by increasing their knowledge or influencing their attitudes. The implementation of health-promoting behaviours such as exercise, together with avoidance of health- risk behaviours such as alcohol consumption, drug use, unprotected sex, smoking, and obesity are essential to reduce the risk of cancer, sexually transmitted diseases (STDs) or unwanted pregnancy, cardiovascular disease and type 2 diabetes [5].

The focus of most current research strategies is to identify ways to combat health risk behaviour. For instance, Pharaoh et al. [6] identified specific life skill domains, as determined by the Life Effectiveness Questionnaire (LEQ), which were positive predictors of engagement in health risk behaviour among South African adolescents. It is therefore postulated that by providing youth with the necessary education and life skills in conjunction with targeting critical contextual factors that influence youth behaviour, high- risk behaviour could be reduced or prevented [7].

### Health risk behaviour: overview and definition

Risky behaviour can be described as; “behaviour that is either physically or emotionally dangerous, or contributes to development problems for young people involved.” [4]. According to Trimpop [8], risky behaviour or risk-taking behaviour is defined as “any consciously, or non-consciously controlled behaviour with a perceived uncertainty about its outcome, and about its possible benefits, or costs for the physical, economic or psycho-social well-being of oneself or others.” [9].

Health-risk behaviour is a leading contributor to morbidity and mortality among children, which are established during childhood and extend into adulthood [10]. Participation in risk-taking behaviour such as substance use decreased physical activity, and risky sexual behaviour practices usually make their debut during adolescents and thus have primarily been studied during that phase of development [11]. However, when these risk behaviour activities are present in children and young people, it could endanger their normal development [4]. The use of alcohol among young children and young people could lead to alcohol-related injuries, academic, behavioural, and relationship problems, as well as the development of lifestyle diseases [4]. Thus, engagement in these types of activities could compromise well-being, health, and life-course development, which could further contribute to the global burden of disease [10]. Health–risk behaviour includes: (a) activities that contribute to unintentional injury and intentional injury and violence; (b) tobacco use; (c) alcohol and other drug use; (d) sexual behaviour that contributes to unintended pregnancy and sexually transmitted diseases (STDs) including human immunodeficiency virus (HIV); (e) dietary practices and (f) physical inactivity according to the Centre of Disease Control [12].

### Health-risk behaviour: prevalence

The high prevalence of youth engaging in risky behaviour that may influence their development in South Africa remains a significant concern from a public health perspective.

In South Africa, the first youth risk behaviour survey was conducted in 2002, and the same study was repeated in 2008 [1]. The 2008 study surveyed 10,000 learners between grades 8 and 11; and found that 38% of the learners were engaged in sexual activity. Furthermore, 20% were classified as obese, 21% had considered suicide or attempted suicide, and 30% were smokers [1]. .Almost 50% of learners had reported having drunk alcohol, and 35% reported having drunk alcohol in the past month.

In the Paarl region alone, a study by Pharaoh et al. [6] surveying a little over 1000 adolescents between the ages of 13–18 years reported engaging in behaviours such as using tobacco (*n* = 660; 64.3%), consumed alcohol (*n* = 510; 49.65%), have used dagga (*n* = 251; 24.4%), have used cocaine (*n* = 24; 2.5%) were sexually active (*n* = 258; 25.1%), were physically inactive (*n* = 398; 38.8%) and other behaviours that contribute to unintentional injury and violence [6]. Despite the best efforts being made to educate the youth on the harmful effects of health-risk behaviour, it is still evident that South African adolescents were engaging in risky behaviour at an alarming rate with little concern about the consequences of their actions [6].

### Health-risk behaviour: theoretical framework

#### The theory of reasoned action

The theory of reasoned action (TRA) has been widely used as a model for the prediction of behavioural intentions [13]. The scientific bases for the development of the TRA was constructed on the assumption that the behaviours being studied were under full volitional control [13]. The TRA theorizes that behavioural intentions, which are the immediate precursors to behaviour, are a function of salient information or beliefs about the likelihood that performing a particular behaviour will lead to a specific outcome [13].TRA divides the precursors to behaviour into two distinct conceptual sets; behavioural and normative. The behavioural beliefs are said to be the underlying influence on the individual’s attitude towards performing the behaviour, whereas the normative beliefs influence the individual’s attitude towards achieving the behavior [13].

The theory of planned behaviour extends the limitation of simple volitional control by including beliefs regarding the possession of resources and opportunities for performing a given behaviour [13]. Thus, this expansion on the TRA postulates that the more access an individual thinks they have to resources and opportunities, the greater should be their perceived control over the behaviour. Thus, perceived control may be seen as an exogenous variable that has both a direct and indirect effect on behaviour [13].

#### The bio-ecological perspective on human development

Bronfenbrenner and Morris [14] have long argued for an ecological approach to understanding human development. Bronfenbrenner and Morris [14] suggested that human development takes place through a process of progressively more complex reciprocal interaction between an active, evolving biopsychological human organism and the persons, objects, and symbols in its immediate external environment [10]. Thus, to understand human development, humans should be studied in their natural living environments where interactions could occur on a regular basis and over an extended period of time, and not by recreating artificial situations [14]. Enduring interactions are referred to as proximal processes that are the primary engines of development.

#### The Ecological Risk and Protective Theory (ERPT)

The ERPT was developed by Borgenschneider [15] and assimilated the perspectives of the bio-ecological theory of human development [14] and developmental contextualism [16].

The bio-ecological theory postulates that it is of necessity to identify risk and protective processes at several levels of human ecology, including individual, family, peer, school, and community settings [17]. This contextualization of human development evolves the bio-ecological theory into a multi-dimensional model emphasizing the dynamic and reciprocal nature of human development.

Despite a societal mind shift to educate young people on the prevention of risky behaviour, behavioural change is not progressing at the rate needed to change health risk behaviour positively [2]. Combating behaviour, even at an adolescent age, implies that specific behavioural patterns may have already been formed and contextualized by influences from childhood. In South Africa, influences such as growing up in an urban or rural setting and socio-economic status could contribute to the choice’s adolescents make with regards to risky behaviour. Implementation programs developed to address health risk behaviour have traditionally taken aspects like these into account; however, the question is, “Are these programs being implemented too late?”

## Methods

### Study setting

The study will be conducted in the Drakenstein Municipality, which has the town of Paarl at its center. Paarl is a densely populated town with a population of 197,735 (census 2011) [18], located about 59 km from Cape Town city center and categorized as a peri-urban. The municipal census (2011) indicated that a little under a third of the population consisted of young people and adolescence [18]. The Paarl region has 20 primary schools in the area, with approximately 50 learners per class and three classes per grade currently enrolled in grades 4–7. Thus, the estimated learner population is 12,000 learners. The peri-urban categorization of the research setting denotes that both rural and urban characteristics co-exist. The study will consist of a sequential, explanatory mixed-methods approach. A mixed-methods approach incorporates both a quantitative phase and a qualitative phase. Baseline data will be obtained, collated, and analysed quantitatively. Second phase data will be collected using focus group interviews and thus analysed qualitatively. A sequential mixed-methods approach allows the results of one phase of a study to inform the next phase of the study. In this study, the primary investigator (PI) will use the results of the baseline data collected to build questions for the focus group sessions in the second phase of the study.

### Study population

The study population will comprise of various stakeholders, including all grade 4–7 learners currently enrolled in a primary school in the Paarl region, teachers, parents, school nurses and influential community representatives involved in life skills training in the area.

### Sampling

All stakeholders in the region will be invited to participate in the study. Stratified random probability sampling will be used to select three primary schools in the area. This will allow for a maximum learner population of 1800 learners for the first phase risk analysis survey. Nonprobability purposive sampling will be used to select the stakeholders participating in the second phase focus group sessions. Six to eight stakeholders associated with each of the selected schools will make up the focus group.

### Sample size calculation

Sample size calculations are based on a Raosoft Inc. online sample size calculator software. A total of 31 schools in the region, and 50 learners per class with three classes per grade would yield a total sample size of 18,600 learners. Using the Raosoft Inc. software, a minimum sample size was calculated (Margin of error = 5%, Confidence level = 95%, Total population size =18,600 learners, Response distribution = 50%, Recommended minimal sample size = 377 learners). Thus, the stratified sampling of three schools should be enough to achieve the minimum sample required.

### Inclusion criteria

- All grade 4–7 learners currently enrolled with the department of education and attending a primary school in the Paarl region of the Drakenstein Municipality.
- All stakeholders presently involved in life skills development or training including, but not limited to, teachers, parents, school nurses, influential community representatives.

### Exclusion criteria

- Learners with disabilities at special needs schools.
- Any stakeholder who is unable to complete the self-reported questionnaire for any medical reason.

### Study framework (intervention mapping)

Intervention Mapping is not a new health promotion theory framework and was first introduced by Bartholomew, Parce, and Kok [19]. It is a framework that tries to bridge the gap between theories and practice and is a stepwise approach to describe the planned process for theory and evidence-based development, implementation, and evaluation of health promotion interventions [19]. Despite the step-by-step approach, the planning process is iterative and cumulative rather than linear, with programs moving in both directions as new themes and concepts are evolving, and each step depends on the findings of the preceding step [20]. A systematic review by Garba and Gadanya [20] reported that most studies that utilized an intervention mapping framework has found it to be useful in designing disease prevention programs, and has been successfully applied in communicable and non-communicable disease prevention.

### Pre-data collection

Before commencing with data collection, the following activities will be completed by the researcher.

### School visits

Data will be collected at three primary schools in the Paarl region of the Drakensberg municipality. The researcher will schedule appointments with the principals of the schools after the successful registration of ethics for the research project. These visits will aim to determine facility-specific operating procedures. Also, facility friendly methods will be developed in consultation with each facility to facilitate the data collection process.

### Training of research assistants

Three fieldworkers will be recruited and trained to assist with data collection. These three fieldworkers will assist in completing the data collection at all the sites.

### Data collection

#### Phase 1

Phase 1 will consist of a needs analysis assessment. Before the administration of the survey, the PI will be responsible for training the RA. The assessment will be conducted in the form of a self-administered questionnaire. Self-reported Child Health Risk Behaviour Survey (CHRBS) questionnaires will be administered at the included sites in paper format.

The PI and trained RA’s will be available on-site during the administration of the questionnaire to answer any questions that the learners may have. A short description and explanation of what is expected and what the study entails will be done before administration. Learners will be allowed to refrain or exit the survey at any point.

#### Pilot trial: data collection procedure pilot

Before the data collection period, the CHRBS questionnaire data collection procedure will be piloted on an independent group of conveniently selected grade 4 to grade 7 learners.

The pilot would aim to establish if the learners understand the questions and can answer the items in the questionnaires. It will also help the PI determine the time needed for the learners to complete the survey.

The desired outcome would be to enable the PI to optimise the data collection procedure.

#### Phase 2

Phase 2 will consist of semi-structured focus group interviews. Each focus group will consist of 6–8 parents and community members. The PI will conduct the interview in a quiet area using double voice recorders. Open-ended, semi-structured questions will be presented to the group. The PI will facilitate the sessions, allowing everyone to have an opportunity to speak freely. All interviews will be transcribed by a professional and university-approved transcriber.

#### Validity

##### Face validity

All transcripts will be made available to the interviewee to confirm the content of the transcribed script.

##### Content validity

The PI will attend a focus group facilitation workshop and be trained to facilitate the focus group sessions. This will ensure that the content asked is the content gained from the sessions.

#### Phase 3

Phase 3 will consist of a systematic review of the literature on current child or adolescent health risk behaviour prevention strategies. Keywords will be identified and used to search five databases, i.e., PubMed, Cochrane, EBSCOhost, Cinahl, and PEDRO, for literature on the effectiveness of health risk behaviour interventions. All included studies will be critically appraised for quality. If possible, homogenous data will be collated and entered into a meta-analysis to establish effectiveness.

#### Phase 4

Development of a children’s health risk behaviour program. The results of phase 1 and 2 will be compared to the results of the systematic review in phase 3. This will inform the development of the intervention prevention program.

#### Phase 5

**Fig. 1:**
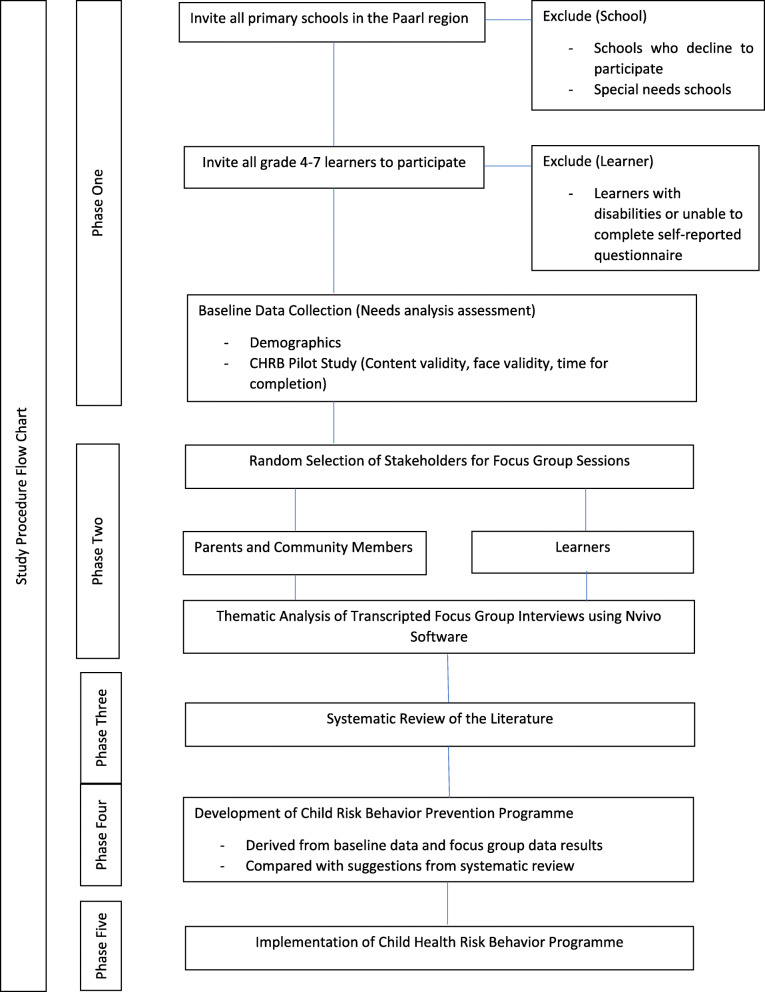
Study Protocol Procedure

Implementation of children’s health risk behaviour prevention program developed in Phase 4 (Fig. 1).

### Tools and data collection methods

#### The child health risk behaviour survey (CHRBS) questionnaire

The CHRBS was developed from the youth risk behaviour surveillance survey and is a 21-item questionnaire covering the domains of potential for unintentional injury, the potential for injury or violence, tobacco use, alcohol, and other drug use, sexual activity, and health practices. The CHRBS was previously developed and published in a study by Riesch et al. [17] and scored a test-retest reliability coefficient of .958 and .977 between a small city and large city sample [17]. The CHRBS will be translated into Afrikaans and adapted for use in the South African context.

#### Data analysis plan

Descriptive statistics, namely means, frequencies, and percentages will be used to analyse the quantitative data where the data is normally distributed. Medians and standard deviations will be used where the data is skewed. Chi-square and t-test will be used for the analysis of variance, and multiple regression analyses will be used to predict health risk behaviour engagement.

Semi-structured interviews and focus groups will be audio-recorded, transcribed verbatim, and anonymised before being coded. Thematic analysis techniques will be used to generate initial codes using the latest available version of NVivo, and these will be grouped to form themes for each cohort.

#### Data management

Once data is collected at any given site, the PI will file the list of learners names (class list), consent forms and anthropometric data in a file which will be securely stored in a locked draw in the physiotherapy department at the University of KwaZulu-Natal, of which only the PI will have access. Anonymization will occur at the time of data collection by assigning codes to all learner’s identifiable information. Once the leaner has been anonymized, only the research codes will be used to identify participant data. The research codes will be linked to the learner identifiable data, but this will be stored in a separate identification database. All electronic data entered into the databases will be stored on a password-protected research laptop, for which only the PI and supervisor will have access to during data collection. A back-up will be made of all electronic databases and stored on a research flash drive, which will be stored in a locked drawer at the physiotherapy department at the University of KwaZulu-Natal, for which only the PI and supervisor will have access.

##### Questionnaires

Self-reported questionnaires will be administered at the included sites using paper copies. Each questionnaire will be coded with a research number, which in turn is traceable to the original learner information, which will be stored in a locked drawer at the physiotherapy department at the University of KwaZulu-Natal, of which only the PI will have access. Data entries will be entered into the research database, which will be developed in consultation with the statistician, and stored electronically on a password-protected research laptop.

##### Focus group session recordings

Small group, semi-structured interviews will be facilitated by the PI at the various data collection sites. The interviews will be recorded on a voice recorder and then transcribed by an independent research assistant. Central and recurring themes will be isolated and used in the formulation and development of the intervention program.

### Ethics approval and consent to participate

Ethical clearance has been granted from the Research Ethics Committees and Higher Degrees Committees of the University of Kwa-Zulu Natal (HSSREC/00001649/2020). Permission to conduct the study has been obtained from the Western Cape Education Department, principals, and governing bodies of the schools involved. The participants will be informed, throughout the research, that their participation is voluntary and that they could choose not to participate or withdraw at any given time during the study without any consequences.

Before the investigation commences, a detailed explanation of the survey will be provided to the learners. All learners will be requested to participate in the study, and all those who have written parent consent (Additional file 1) and have given written assent themselves will be part of the study. Confidentiality and anonymity will be assured to those learners that will be participating. All precautions in relation to COVID19 safety and prevention of infection will be followed.

#### Study outputs and dissemination plan

It is hoped that this study will provide valuable information that will contribute to the re-design of current models of health risk behaviour in the South African public health sector. The proposed audience for the dissemination of the study results will be the study participants, staff members at the research site, colleagues in the School of Health Science and wider UKZN community, peer researchers, and potential funders. In order to facilitate the uptake of evidence from this study into policy and practice, the study results will also be presented to appropriate staff in the Western Cape provincial and national Department of Health, as well as other relevant stakeholders. The proposed methods of disseminating the study results will include public and stakeholder presentations, knowledge translation workshops, as well as the planned publication of results in peer-reviewed journals and presentations at relevant academic conferences.

## Discussion

To the researchers’ knowledge, this is the first study that would attempt to establish a health risk behaviour prevention program in youth and young people in South Africa. Overwhelming evidence exists that adolescents engage in risky health behaviour which may potentially negatively impact their lives. Most current research is focussed on the adolescent age group with the aim of introducing an intervention plan. Unique socio-economic circumstances that South African adolescents are exposed to from a young age like growing up in an urban or rural setting and the massive divide between the rich and the poor means that certain risky health behaviours may have already been formed and contextualised by these circumstances.

This study provides an opportunity to address a gap in the current strategy by developing a prevention program for young people, which could later be supported by further booster programs through their adolescents. It would, therefore, be postulated that if prevention could start in childhood years, it could assist young people and adolescents in making less risky health behaviour decisions. This project would serve as a baseline prevention program that could help in the reduction of risky health behaviour among various communities.

## Supplementary Information

**Additional file 1.**

## Data Availability

Not Applicable.

## Abbreviations

WHO: World Health Organisation
STD: Sexually transmitted disease
LEQ: Life Effectiveness Questionnaire
HIV: Human Immunodeficiency Virus
TRA: Theory of Reasoned Action
ERPT: Ecological Risk and Protective Theory
PI: Primary Investigator
CHRBS: Child Health Risk Behaviour Questionnaire
