# Supplementary material for: Developing a children’s health risk behaviour prevention program targeting grade 4–7 learners in the western cape, South Africa: a study protocol
Source: BMC Public Health. 2021 May 30;21:1021. doi: 10.1186/s12889-021-10968-6 (PMC8166084; doi:10.1186/s12889-021-10968-6)
Supplement: Supplementary file 1 — Additional file 1. [file 12889_2021_10968_MOESM1_ESM.pdf]

Appendix A

ASSENT FORM FOR MINORS

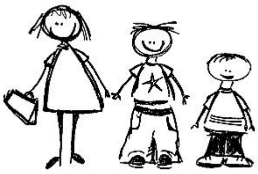

TITLE OF THE RESEARCH PROJECT: The Development of a children's health risk behaviour prevention program targeting grade 4-7 learners in the Paarl Region, South Africa

RESEARCHERS' NAME(S): Kurt John Daniels

RESEARCHER'S CONTACT NUMBER:

What is RESEARCH?

Research is something we do find **NEW KNOWLEDGE** about the way things (and people) work. We use research projects or studies to help us find out more about children and teenagers and the things that affect their lives, their schools, their families and their health. We do this to try and make the world a better place!

What is this research project all about?

We would like to build a program that you can access through your school that will help you in case you are experiencing any challenges or problems to your learning and development.

Why have I been invited to take part in this research project?

You have been invited to participate in this study because you are a learner in grades 4 to 7 in the Paarl region

Who is doing the research?

My name is Kurt John Daniels. I am a doctorate student working for the University of KwaZulu-Natal.

What will happen to me in this study?

You will be asked to complete a series of questions. These questions will be based around your health and your everyday behaviour

Can anything bad happen to me?

No! Nothing bad will happen to you during the study. We are concerned about your health and well being.

Will anyone know I am in the study?

Taking part in this study is entirely voluntary. If you choose to be part of this study, you may withdraw at any time without consequences of any kind. You are not obligated to answer all the questions. Participating in this study does not mean that you are giving up any of your legal rights. Failure to cooperate with researchers will result in termination from the program. Your identity will be kept private throughout the entire process (no personal details of you will be revealed at any time to anyone but the research team and our supervisor). The records of this study will be kept private, and all the completed questionnaires will be stored in the Department of Physiotherapy for five years. After that, it will be destroyed.

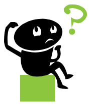

Do you understand this research study and are you willing to take part in it?

YES NO

Has the researcher answered all your questions?

YES NO

Do you understand that you can STOP being in the study at any time?

YES NO

___________________________ ______________________

Signature of Child Date

CHILD HEALTH RISK BEHAVIOR SURVEY (CHRBS)

INTRODUCTION

Child Health Risk Behavior Survey

This survey is about health behaviour. It has been developed so you can tell us what you do that may affect your health. The information you give will be used to improve health education for young people like yourself.

DO NOT write your name on this survey. The answers you give will be kept private. No one will know what you write. Answer the questions based on what you really do.

Completing the survey is voluntary. Whether or not you answer the questions will not affect your grade in this class. If you are not comfortable answering a question, just leave it blank.

The questions that ask about your background will be used only to describe the types of students completing this survey. The information will not be used to find out your name. No names will ever be reported.

Make sure to read every question. Fill in the ovals completely. When you are finished, follow the instructions of the person giving you the survey.

Thank you very much for your help.

Section A: Some information about yourself

1. How old are you?

A. 9 years old or younger
B. 10 years old
C. I1 years old
D. 12 years old
E. 13 years old
F. Older than 13 years

2. Are you a:

A. Girl
B. Boy

3. In **what grade are you?**

A. 4th grade
B. 5th grade
C. 6th grade
D. 7th grade

4. What is your race group?

A. Black
B. Coloured
C. Indian
D. White
E. Other

6. How tall are you without your shoes on?
Directions: Write your height in the spaces below.

Height

Metres_ _ _ _ _ _ _

Centimetres_ _ _ ___________

7. How much do you weigh without your shoes on?
Directions: Write your weight in the spaces below.

Weight

Kilograms ____________

Children's Health Risk Behaviors Scale (2020)

Have any of your friends	Have you ever
1. Ridden a bicycle or used a skateboard, scooter or skates without a helmet?<br>Never Sometimes Always Don't Know	Ridden a bicycle or used a skateboard, scooter or skates without a helmet?<br>Never Sometimes Always Don't Know
2. Ridden in a car without a seat belt?<br>Never Sometimes Always Don't Know	Ridden in a car without a seat belt?<br>Never Sometimes Always Don't Know
3. Ridden with a driver who had been drinking?<br>Never Sometimes Always Don't Know	Ridden with a driver who had been drinking?<br>Never Sometimes Always Don't Know
4. Swam in a river, canal or dam where it is unsafe?<br>Never Sometimes Always Don't Know	Swam in a river, canal or dam where it is unsafe?<br>Never Sometimes Always Don't Know
5. Carried a weapon such as a gun, knife, blade or punga without adult permission<br>Never Sometimes Always Don't Know	Carried a weapon such as a gun, knife, blade or punga without adult permission<br>Never Sometimes Always Don't Know
6. Felt unsafe at school?<br>Never Sometimes Always Don't Know	Felt unsafe at school?<br>Never Sometimes Always Don't Know
7. Had something stolen?<br>No Yes Don't Know	Had something stolen?<br>No Yes Don't Know
8. Stolen something?<br>No Yes Don't Know	Stolen something?<br>No Yes Don't Know
9. Been in a physical fight?<br>No Yes Don't Know	Been in a physical fight?<br>No Yes Don't Know
10. Thought about killing his/her self?<br>No Yes Don't Know	Thought about killing his/her self?<br>No Yes Don't Know
11. Smoked a cigarette, hubbly bubbly (Okka pyp) or vape, even one or 2 puffs?<br>No Yes Don't Know	Smoked a cigarette, hubbly bubbly (Okka pyp) or vape, even one or 2 puffs?<br>No Yes Don't Know

12. Smoked a whole cigarette?<br>No Yes Don't Know	Smoked a whole cigarette?<br>No Yes Don't Know
13. Chewed tobacco or used snuff or dip?<br>No Yes Don't Know	Chewed tobacco or used snuff or dip?<br>No Yes Don't Know
14. Drank beer, wine, or liquor without adult permission?<br>No Yes Don't Know	Drank beer, wine, or liquor without adult permission?<br>No Yes Don't Know
15. Smoked dagga (marijuana, grass, pot)?<br>No Yes Don't Know	Smoked dagga (marijuana, grass, pot)?<br>No Yes Don't Know
16. Inhaled/breathed fumes (such as glue) to get high?<br>No Yes Don't Know	Inhaled/breathed fumes (such as glue) to get high?<br>No Yes Don't Know
17. Used other illegal drugs such as cocaine, tik, or drugs you inject with a needle?<br>No Yes Don't Know	Used other illegal drugs such as cocaine, tik, or drugs you inject with a needle?<br>No Yes Don't Know
18. Gone out with' a girl or boy friend?<br>No Yes Don't Know	Gone out with' a girl or boy friend?<br>No Yes Don't Know
19. Held hands or put arms around girl/boy friend?<br>No Yes Don't Know	Held hands or put arms around girl/boy friend?<br>No Yes Don't Know
20. Kissed girl/boy friend?<br>No Yes Don't Know	Kissed girl/boy friend?<br>No Yes Don't Know
21. What time do your friends usually go to bed on school nights?<br>What time do your friends usually get up on school mornings?	What time do your friends usually go to bed on school nights?<br>What time do your friends usually get up on school mornings?

22. Tried to lose weight very quickly?<br>No Yes Don't Know	Tried to lose weight very quickly?<br>No Yes Don't Know
23. Do exercises (or sports) at least 20 minutes 3 times/week?<br>Never Sometimes Always Don't Know	Do exercises (or sports) at least 20 minutes 3 times/week?<br>Never Sometimes Always Don't Know
